# Exploring nonlinear phenomena in animal vocalizations through oscillator theory

**DOI:** 10.1098/rstb.2024.0015

**Published:** 2025-04-03

**Authors:** Marta del Olmo, Christoph Schmal, Hanspeter Herzel

**Affiliations:** ^1^ Humboldt-Universität zu Berlin Institute for Theoretical Biology, Berlin, Germany

**Keywords:** animal vocalizations, nonlinear phenomena, oscillator theory, attractors, bifurcations, adaptive functions

## Abstract

Animal vocalizations comprise a rich array of complex sounds that exhibit nonlinear phenomena (NLP), which have fascinated researchers for decades. From the melodic songs of birds to the clicks and whistles of dolphins, many species have been found to produce nonlinear vocalizations, offering a valuable perspective on the mechanisms underlying sound production and potential adaptive functions. By leveraging on the principles of oscillator theory and nonlinear dynamics, animal vocalizations, which are based on coupled oscillators, can be described and conveniently classified. We review the basic ingredients for self-sustained oscillations and how different NLP can emerge. We discuss important terms in the context of oscillator theory: attractor types, phase space, bifurcations and Arnold tongue diagrams. Through a comparative analysis of observed NLP and bifurcation diagrams, our study reviews how the tools of nonlinear dynamics can provide insights into the intricate complexity of animal vocalizations, as well as into the evolutionary pressures and adaptive strategies that have shaped the diverse communication systems of the animal kingdom.

This article is part of the theme issue, ‘Nonlinear phenomena in vertebrate vocalizations: mechanisms and communicative functions’.

## 1. Introduction

Animal vocalization constitutes an extremely complex system, often exhibiting nonlinear phenomena (NLP). Early studies in the field showed that different dynamical behaviours, such as subharmonics, biphonation, frequency jumps and deterministic chaos, can arise from animal vocalizations [1–6]. These are all known as NLP. The breakthrough of nonlinear dynamics and chaos theory from the twentieth century ignited a surge of interest in understanding the intricacies of animal vocalizations, driving experimental researchers to collect and classify hundreds of animal calls characterized by NLP. These vocalizations, recorded from a wide variety of animals—including non-human primates, canids, deer, farm animals, frogs, rodents, birds and dolphins [1,6–14]—provided valuable insights into the mechanisms of sound production and the potential adaptive functions of these NLP.

From a physical perspective, sound production can be understood through the concept of coupled nonlinear oscillators [15,16]. Coupled oscillators refer to systems where multiple oscillating components influence each other, leading to complex sound (or other rhythmic) patterns. In animal vocalization, these oscillators include vocal folds, left and right syringes, vocal tract resonators or supraglottal structures such as ventricular folds. Consequently, oscillator theory [17–19] provides an appropriate framework for describing and classifying animal vocalizations. In this review, we discuss the basics of self-sustained oscillations and NLP. We start by revisiting different types of attractors and introducing the concept of phase space. One of the key tools in the analysis of NLP are bifurcation diagrams, which help in understanding the complexities of animal sounds and how sounds change as system parameters slowly vary. We thus examine how bifurcations occur as system parameters change, and what the implications of these bifurcations are for vocalizations. The complexity of coupled oscillators is finally visualized through the so-called Arnold tongue diagrams: these two-dimensional bifurcation plots offer a clear visualization of the possible outcomes of coupled oscillator systems and enable testable predictions in the field of animal bioacoustics, ultimately contributing to a better understanding of complex vocalizations.

## 2. From transients to attractors

The generation of sound is based on vibrating structures such as tissues, membranes and columns of air. Reptiles, amphibians and mammals all have a larynx, a voice box at the top of the throat that protects the airways. Folds of tissue there—the vocal cords or vocal folds—can vibrate to enable humans to talk, pigs to grunt and tigers to roar. The melodious call of many birds, on the other hand, comes from a different organ located within their chests at the caudal end of the trachea: a special voice box termed the syrinx [20–22]. Additional physiological structures can also contribute to phonation, including vocal tract resonators or air sacs [5].

Typically, when excited, these oscillating structures can induce damped oscillations, similar to the vibrations of a piano string after being struck by a hammer. During this vibration, energy losses (commonly termed dissipation) will lead to decreasing amplitudes and ultimately to steady states. Such damped oscillations are also termed *transients*, because it is a dynamical behaviour which will evolve—after some time—to an asymptotic state also called *attractor*. In the case of damped oscillations, the attractor is said to be stable focus, stable steady state, fixed point or equilibrium.

**Figure 1.**
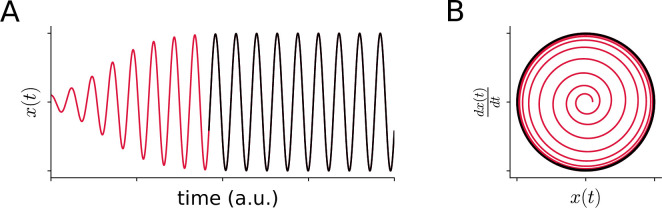
Transients and limit cycles. This figure illustrates the behaviour of initial conditions as they evolve over time in a time series (*A*) or in phase space (*B*). The red trajectories represent transients that approach the attractor, a stable limit cycle.

However, to obtain sustained phonation, energy supply has to compensate for omnipresent dissipation. According to the established myoelastic–aerodynamic theory [16], the airflow and wave-like vibrations of vocal folds allow energy transfer from respiration to the vocal folds. Similar mechanisms drive sustained vocalization in syringes in birds [23], infrasound generation in elephants [24], vocalization of whales [25] and even purring of cats [26], thus contributing to phonation in these animals. When oscillations become self-sustained with autonomously controlled periods (or frequencies) and amplitudes, they are termed limit cycles. Limit cycles are another type of attractor, as any perturbation that pushes the system out of the stable cycle will eventually dampen out (see red transient in figure 1), and the trajectory of the perturbed variable will be asymptotically ‘attracted’ back to the limit cycle (black in figure 1). The generation of stable limit cycles is controlled by nonlinearities of tissue vibrations and airflow, and stable amplitudes are achieved by a balance of energy supply and dissipation.

To illustrate how the state of a dynamical system changes with time, phase space trajectories become useful. In dynamical systems theory, a phase space is a space in which all possible states of a dynamical system are represented, with each possible state corresponding to one unique point in phase space. A phase space may contain a great number of dimensions: in the field of animal vocalization, possible dimensions could be the displacement of the left and right vocal folds, glottal volume flow or sound pressure [27].

**Figure 2.**
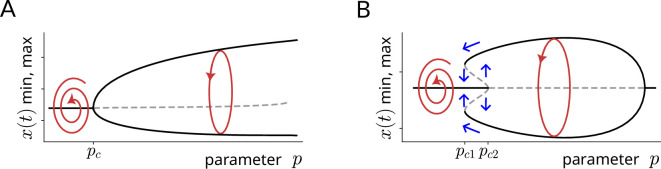
Onset of self-sustained limit cycle oscillations owing to increasing parameter p (Hopf bifurcation). (A) Onset of limit cycle oscillations through a supercritical Hopf bifurcation: as *P* gradually increases, the system transitions from damped oscillations (red trajectory for $p<p_{c}$) to self-sustained oscillations (red limit cycle for $p>p_{c}$). (B) Onset of limit cycle oscillations through a subcritical Hopf bifurcation: in this type of bifurcation, large amplitude oscillations suddenly emerge as p is increased (at $p=p_{c2}$). However, when p is decreased, the limit cycle vanishes at a lower critical value $p_{c1}$, rather than the value at which it emerged. Consequently, there is a range of parameter values for which stable steady states and limit cycles coexist, indicating hysteresis. Amplitude jumps are indicated by blue arrows.

In a two-dimensional phase space, stable steady states or limit cycles are possible attractors. In the case of stable steady states (figure 2A), such as in cases of aphonia, trajectories (red) asymptotically approach the fixed point (black) in the phase portrait. In the case of attracting limit cycles (figure 2B), transients (red) are attracted not to a point but to an oscillating cycle (black). More complex attractors, like tori (figure 2C) or chaotic attractors (figure 2D) require at least three phase space dimensions [28]. This dimensionality is essential because, in deterministic dynamical systems, each point in the phase space is associated with a unique direction and speed of evolution. Since trajectories of an attractor in phase space follow a single, specific path, intersections are not allowed. In two dimensions, a chaotic or toroidal trajectory would inevitably self-intersect after some time, contradicting the fundamental rule of uniqueness. To avoid this, systems exhibiting such complex behaviours require at least three phase space dimensions. A two-torus is represented in figure 2C: such attracting structures are generated by two oscillations of different frequencies that have an irrational ratio (i.e. $f_{1}/f_{2}\neq p/q$ for any integers p and q). This irrational frequency ratio leads to quasi-periodic motion, whereby the system’s behaviour never returns to the same point. If the frequencies $f_{1}$ and $f_{2}$ have an integer ratio p/q, the dynamics are not toroidal but instead the attractor is called a folded limit cycle or said to be frequency-locked. Finally, chaotic structures are aperiodic and exhibit intrinsic instability that leads to noise-spectral components (figure 2E). Nevertheless, chaos can be generated by strictly deterministic mechanisms (e.g. by coupled nonlinear oscillators). In contrast to pure noise, deterministic chaos often contains residual periodic components (figure 2E) perceived as rough-sounding vocalizations such as screams or roars [29,30].

Attractors and NLP can also be visualized in a spectrogram, i.e. in a representation of the spectrum of frequencies of a signal as it varies over time (figure 2E). If a limit cycle sound at a particular frequency $f_{0}$ emerges from silence, that is known as *onset of oscillations* (note the harmonics occurring at whole-number multiples of the fundamental frequency $f_{0}$ in figure 2E). If the sound’s frequency changes, this will be illustrated in the spectrogram as a *jump* to a second frequency (and its harmonics). Sound recordings might be more complex and contain *subharmonics*, which are characterized by the presence of subharmonic tones below the fundamental frequency; or even *chaotic*, with noise-like spectral components. Chaos can be caused by vocal folds vibrating in a highly irregular manner, as in rough barks or roars, which effectively ‘smears’ the harmonics introducing variable amounts of broadband noise to the spectrogram. Some animals such as penguins, elks or killer whales can produce two audible frequencies at the same time [31], a phenomenon known as *biphonation* that results in toroidal attractors (as in figure 2C) in phase space. NLP encompass all these acoustic events.

In other theoretical studies, attractors and NLP have been classified using Lyapunov exponents and fractal dimensions [32–34]. In many cases, narrowband spectrograms are a rather helpful technique to identify attractors, especially for detecting independent frequencies in biphonation [1,7,35,36].

## 3. Bifurcations owing to slowly varying parameters

Sound production in animal vocalization involves several key parameters, including subglottal pressure, muscle tension, vocal tract geometry and the viscoelastic properties of tissues [27,37]. Together, these parameters play a crucial role in shaping the dynamics of voice or animal call production. From a physical point of view, the dynamics of sound production can be described through transients and attractors. Systematic variation of parameters can induce qualitative changes in the dynamics (i.e. bifurcations) and alter the type of attractor that dominates the vocalization. In this section, we discuss the most important types of bifurcations: Hopf bifurcation for phonation onset, period-doubling bifurcations for subharmonics and secondary Hopf bifurcations generating biphonation.

### (a) Phonation onset as Hopf bifurcations

**Figure 3.**
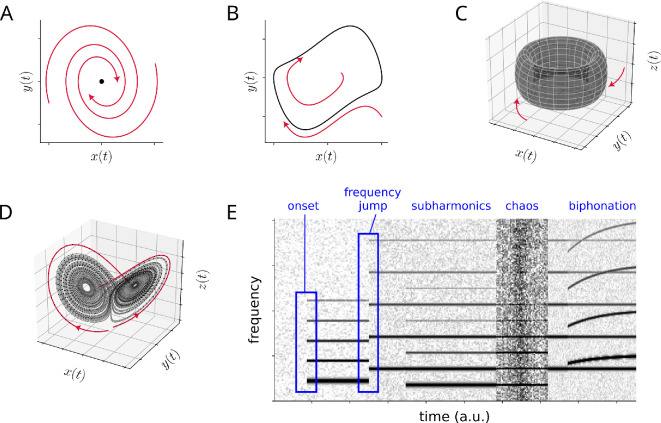
Schematic illustration of attractor types in phase space and spectrograms. Shown are various types of attractors in phase space and their corresponding spectrograms. (A) Stable steady state; (B) stable limit cycle; (C) two-frequency torus; (D) chaotic attractor. Perturbations that kick the system out of its attractor will ‘dampen out’ (red trajectories, exemplifying transients) and the system will return back to the attractor (black trajectories in panels A–D). (E) Sketch of a spectrogram showing various NLP, including the onset of vocalization, frequency jumps, subharmonics, chaos and biphonation. Each type of attractor has a distinctive signature that can be visualized in the spectrogram. Note the harmonics of the fundamental frequency $f_{0}$ appearing at multiples of $f_{0}$.

The transition from damped oscillations to self-sustained limit cycle oscillations is termed a Hopf bifurcation [28,38]. There are two types of Hopf bifurcations relevant to phonation onset: supercritical and subcritical, representing soft and hard onset of oscillations, respectively [39]. Supercritical Hopf bifurcations are characterized by a smooth transition from weakly damped oscillations to a limit cycle (shown in red in figure 3A) as the parameter p reaches a critical value $p_{c}$. As p gradually increases, oscillations change from damped (see red trajectory spiralling to the fixed point in figure 3A for $p<p_{c}$) to self-sustained (red limit cycle in figure 3A for $p>p_{c}$), which grow in amplitude.

By contrast, the subcritical Hopf bifurcation exhibits sudden amplitude jumps. When the parameter p increases beyond a critical value $p_{c2}$, large-amplitude oscillations emerge (figure 3B). When p is decreased, the limit cycle does not disappear at the same value at which it emerged (i.e. $p=p_{c2}$): instead, it vanishes at a lower critical value $p_{c1}$, returning to a stable steady state. A notable feature of the subcritical Hopf bifurcation is the ‘gap’ between the parameter values determining the onset and offset of oscillations. Such a phenomenon is known as *hysteresis* [40,41], and implies that a kind of memory is present in the system, with a steady state and a limit cycle coexisting for parameter values between $p_{c1}$ and $p_{c2}$. Differences in the onset and offset of phonation have been observed in excised larynx experiments [42] and in high-speed recordings of patients [43]. Additionally, hysteresis has been observed in transitions between chest voice and falsetto, both in excised larynx experiments [44] and in computer simulations [45].

### (b) Period-doubling bifurcations generate subharmonics

**Figure 4.**
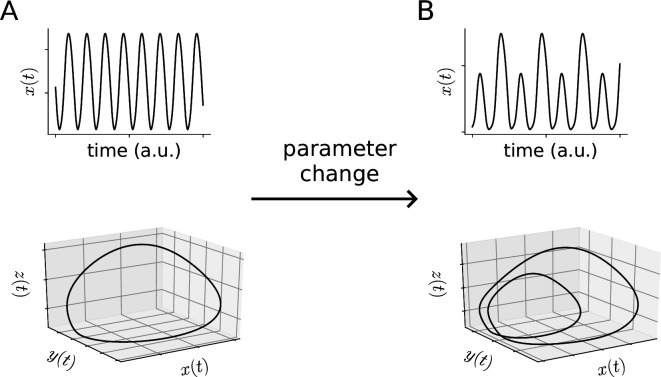
Period doubling bifurcation induced by parameter changes. If a stable limit cycle (A) undergoes a period-doubling bifurcation, the resulting periodic trajectory (B) will exhibit alternating amplitudes and will have double the period of the original cycle. Periodic trajectories are shown as time series (top row, note the alternating amplitudes) and in phase space (bottom row, note the ‘folding’ of the limit cycle).

Beyond limit cycles, more complex attractors are possible in the dynamics of phonation (figure 2E). Another type of bifurcation point generating more complex time series is known as the period-doubling bifurcation [28,46]. Here, parameter changes induce a new periodic trajectory to emerge from the original oscillation—the new one having double the period of the original, with amplitude alternating from small to large, resulting in folded limit cycles in phase space (figure 4). In the spectral domain, period-doubling leads to the emergence of a new peak at half of the fundamental frequency $f_{0}$ and a new stack of peaks (or bands in a spectrogram) interspersed between the previous harmonics, thus resembling frequency halvings or octave jumps. Such period-doubling cascades and subharmonics are frequently found in coupled vibrations of vocal folds and ventricular folds [47–49] or nonlinear source–filter interactions [50]. These observations are also associated with special singing techniques (strohbass), with throat singing or with the vocal fry register. Interestingly, the vocal fry register is used by some species of whales to generate powerful echolocation clicks [51].

Many nonlinear dynamical systems found in nature exhibit a transition from periodic to chaotic motion as some control parameter is varied. There are various known mechanisms by which chaotic motion can arise from periodic motion, but a transition via an infinite series of period-doubling bifurcations (known as period-doubling cascade) is among the most commonly occurring of these mechanisms [28,46].

### (c) Secondary Hopf bifurcations lead to biphonation and modulations

Secondary Hopf bifurcations play an important role in the development of complex vocalizations and NLP in the animal kingdom. These bifurcations occur when changes in system parameters cause an existing oscillation to be complemented by a new, independent frequency. As a result, a two-dimensional torus attractor is formed (figure 2C). In the spectral domain, this bifurcation is observable as new stacks of peaks (or bands) alongside the original harmonics. When both of these frequencies fall within the audible range, the resulting phenomenon is known as biphonation [52,53].

In bird bioacoustics, the production of two independent pitches from the left and right syrinx is an example of two-voice phenomena [4,8]. This capability can function as an individual recognition system, as seen in penguins [4], and is thought to have evolved alongside the loss of territoriality [4]. Interestingly, even a single larynx can biphonate under certain circumstances [8]. Moreover, slow amplitude and frequency modulations can also form tori, which are typically represented in spectrograms as sidebands of existing harmonics [54,55]. These modulations add further complexity to the study of animal vocalizations, highlighting the intricate dynamics of sound production in the animal kingdom.

So far, we have discussed the most relevant bifurcations of limit cycles owing to variations of control parameters, which in the realm of bioacoustics could be driving pressure, muscle tension or asymmetry. In the following section, we expand our approach to two-dimensional bifurcation diagrams of coupled oscillators.

## 4. Coupled oscillators and Arnold tongue diagrams

**Figure 5.**
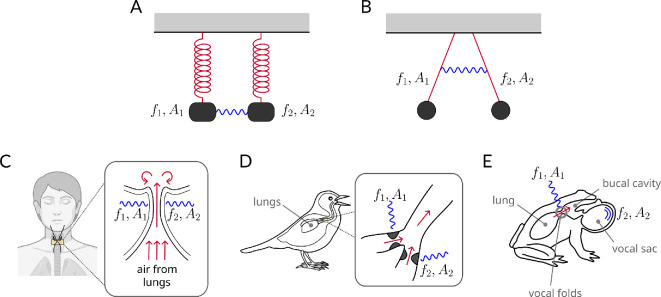
Examples of coupled nonlinear oscillators in physics and biology: (A) coupled springs or (B) pendula. (C) Left and right vocal folds. (D) Left and right syringes in birds. (E) Vocal fold oscillators and resonators. $f_{1}$ and $f_{2}$ represent the frequencies of the two oscillating structures, while $A_{1}$ and $A_{2}$ denote their respective amplitudes.

In 1665, the great Dutch physicist Huygens observed that two identical pendulum clocks, weakly coupled through a beam, quickly synchronized to the same period and amplitude. He watched them for hours, noting that they never fell out of sync, and when he tried disturbing them, they soon regained synchrony. Huygens’ serendipitous observation marked the beginning of a new sub-branch of mathematics: the theory of coupled oscillators. Coupled oscillators can be found throughout non-living systems (e.g. pendula or masses on a spring, figure 5A,B), but they are especially remarkable in living things: pacemaker cells in the heart [46], insulin-secreting cells in the pancreas [56,57], circadian clocks [58,59] or in the generation of sound.

Because it is normally a pair of vibrating tissues or structures (sometimes even more) that generates phonation in the animal kingdom, coupled oscillators are ubiquitous in bioacoustics. Examples are left and right vocal folds [43] (figure 5C), left and right syrinx in birds [60] (figure 5D) and additional oscillators like ventricular folds, vocal membranes [10] and air sac resonators [5,61] (figure 5E), all of which contribute to the complex production of vocalization and NLP in various species.

Coupled oscillators are defined by their intrinsic frequencies and amplitudes, with the coupling strength determining their interaction. The dynamics are primarily influenced by the frequency ratio and coupling strength. When the frequencies are similar and the coupling is strong, the oscillators synchronize, leading to stable vocalizations. However, if the frequencies are mismatched or coupling is weak, the system can display more complex behaviours, such as tori, frequency locking or chaotic dynamics. This section reviews these behaviours and explains how Arnold tongue diagrams aid in understanding NLP in animal bioacoustics.

### (a) Synchronization for $f_{1}$ : $f_{2}$ close to 1 : 1

When two oscillators have similar frequencies $f_{1}$ and $f_{2}$, even weak coupling can lead to synchronization [18,38], and consequently to both oscillators ticking with the same frequency (i.e. 1 : 1 synchronization). For instance, mechanical and aerodynamic coupling of slightly asymmetric vocal folds often results in regular, synchronized vibrations. This synchronization is typical of healthy human phonation and in normal calls in animals. However, asymmetries such as those caused by vocal fold paralysis or desynchronization of vibration modes [62–65] can disrupt this 1 : 1 frequency locking.

### (b) Toroidal oscillations for detuned oscillators and weak coupling

If the coupling between oscillators is too weak to synchronize two independent detuned frequencies $f_{1}$ and $f_{2}$, the system can exhibit independent oscillations known as tori or toroidal rhythms (figure 2C). As previously discussed, these rhythms are characterized by the presence of two or more independent frequencies that do not lock together (i.e. $f_{1}/f_{2}\neq p/q$) but rather coexist, leading to more complex acoustic phenomena. These toroidal rhythms are referred to by various terms, depending on their manifestation and the context in which they are observed, including biphonation, two-voice phenomena, modulation or beating.

Biphonation occurs when two distinct fundamental frequencies are simultaneously produced, resulting in a sound that contains two independent tonal components. This can often be seen in spectrograms as two separate sets of harmonics, each corresponding to one of the fundamental frequencies (figure 2E). This phenomenon is particularly notable in some bird species, such as penguins [4], in killer whales [66] and in certain human vocal techniques [67–69].

Modulation, on the other hand, involves the interaction of these frequencies, where one oscillator modulates the other, leading to the appearance of additional frequencies known as sidebands. These sidebands are typically seen as peaks in the spectrogram that are symmetrically spaced around the main frequencies. Beating is another manifestation of weakly coupled oscillators with detuned frequencies. It occurs when two close but not identical frequencies interfere with each other, producing a fluctuating amplitude pattern that can be perceived as a beating sound. This beating can be visualized in spectrograms as periodic variations in the amplitude of the spectral peaks.

### (c) Frequency locking for $f_{1}$ : $f_{2}$ close to p:q

Apart from 1 : 1 synchronization or toroidal oscillations, rhythms can synchronize at frequency ratios other than 1 : 1, a phenomenon well-studied in locomotion [46,70,71] and other biological systems such as the cardio-respiratory system. For example, fish fin movements might be coordinated with ratios such as 1 : 2, 2 : 3 or 4 : 3 [70]. In some cases, heartbeats and respiration exhibit 4 : 1 frequency locking [72,73], meaning that there are four heartbeats during one respiration cycle. These synchronization patterns, found when frequency ratios $f_{1}$ : $f_{2}$ are close to small integer rations p:q, produce attractors termed folded limit cycles (figure 4), which complete their cycle after several loops. In spectrograms, p:q frequency locking appears as subharmonic bands (figure 2E), often at halves or thirds of the original harmonic stack. Of note, distinguishing between biphonation and p:q frequency locking can sometimes be challenging and requires long and stationary recordings [1,7,35,54,73].

### (d) Deterministic chaos

In addition to folded limit cycles and tori, coupled nonlinear oscillators can also produce a more complex type of dynamics: chaotic attractors (figure 2D). Deterministic chaos is characterized by non-periodic and irregular rhythms and a broadband spectrum. Perceptually, these sounds are rough, harsh or creaky, such as newborn cries or tiger roars, with abrupt onsets and offsets of chaotic episodes in spectrograms. Unlike turbulence in fricatives, chaotic desynchronization of couples oscillators often retains residual spectral components, including some harmonics and subharmonics (figure 2E).

Fricatives are a type of consonant sound produced by forcing air through a narrow constriction in the vocal tract, causing turbulent airflow. Common examples of fricatives in English include the sounds of ‘s’ in ‘see’ and ‘f’ in ‘fish’. The noise generated in fricatives is generally broad-spectrum and lacks the harmonic structure found in voiced sounds, including chaotic calls. By contrast, chaotic desynchronization in coupled oscillators, while also producing broadband noise, often includes distinct spectral components that can be traced back to the underlying oscillatory behaviour of the vocal folds. This difference is crucial for understanding the nature of chaotic sounds in bioacoustics.

Chaotic dynamics have been observed in computer simulations of vocal fold vibrations [27,43,45,60,63] and in excised larynx experiments [24,26,42,44,74,75]. These studies confirm that coupled oscillators are a dominant source of many noisy screams, shrieks and roars in animal communication [3,76].

### (e) Arnold tongue diagrams arrange attractors of coupled oscillators

**Figure 6.**
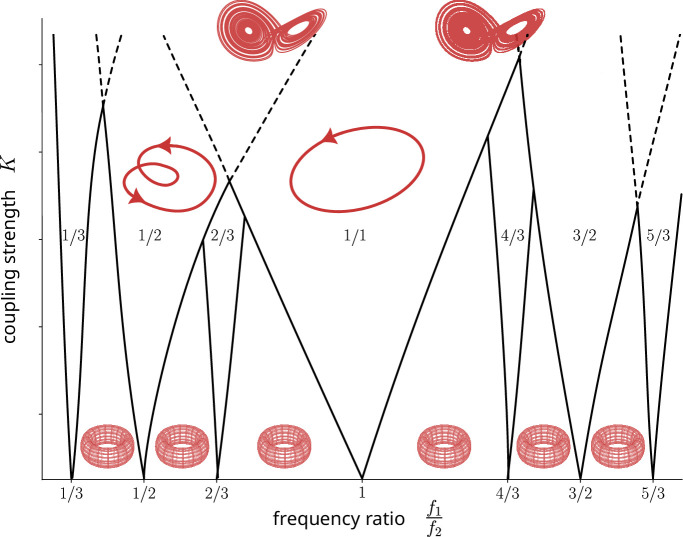
Two-dimensional bifurcation diagram of coupled nonlinear oscillators. This diagram illustrates the behaviour of coupled oscillators as a function of their frequency ratio p/q and the coupling strength K. Arnold tongues refer to the triangular frequency locking regions in the parameter space, and the numbers attached to each region describe the frequency ratio for the entrainment such as 1 : 3, 1 : 2, 2 : 3 or 1 : 1. Between these regions, toroidal rhythms can occur for weak coupling K. Dashed lines indicate where tongues start to overlap, indicating the coexistence of different limit cycles. As the coupling strength K increases, period-doubling cascades can lead to deterministic chaos.

Arnold tongue diagrams provide a structured view of the diverse attractors and bifurcations in coupled nonlinear oscillators. By plotting the frequency ratio $f_{1}/f_{2}$ on the x-axis and coupling strength K on the y-axis, the different attractor types can be identified: 1 : 1 synchronization, p:q frequency locking, torus rhythms and deterministic chaos (figure 6).

In these diagrams, a broad, almost triangular region represents 1 : 1 synchronization. This region expands as the coupling strength K increases. Practically, this means that two similar frequencies $f_{1}$ and $f_{2}$ can synchronize in a 1 : 1 fashion with weak coupling. However, as the frequencies detune, stronger coupling is required to maintain this 1 : 1 synchronization. Thinner tongues in the diagram depict other frequency locking ratios p/q, such as 1 : 2, 2 : 3, 4 : 3 and 3 : 2, corresponding to folded limit cycles with subharmonics. For large q values, the Arnold tongues become very thin, explaining why most subharmonics appear at halves and thirds of the harmonics.

When the coupling strength K is weak, broad areas without simple frequency locking emerge, indicative of independent oscillations or toroidal rhythms (biphonation or modulations). As coupling increases, these tori diminish, leading to either frequency locking or chaotic behaviour if K becomes large. From the plot in figure 6, it also becomes evident that Arnold tongues sometimes overlap at high values of coupling strength. In these scenarios, bifurcations such as frequency jumps with hysteresis, period-doubling and transitions to deterministic chaos become common.

The universal features of Arnold tongue diagrams have been observed in diverse systems, including fish fin locomotion [70], heartbeats with ectopic pacemakers [77], respiration and ventilation [46], entrainment of circadian clocks [78–80] and the segmentation clock [81]. These observations highlight the applicability of oscillator theory and bifurcation diagrams of coupled oscillators as a robust framework for understanding NLP in animal bioacoustics. By providing insights into how different oscillatory behaviours emerge and interact, these Arnold tongues offer a valuable tool for researchers studying the complex dynamics of vocal production and other biological systems.

In summary, bifurcation diagrams of coupled oscillators provide an appropriate framework for understanding NLP in animal bioacoustics. Studying the interactions among oscillators is crucial in order to gain insights into how animals produce a wide range of vocal signals, from harmonious birdsongs to some chaotic mammal calls, and into the evolution of communication strategies. These phenomena highlight the diversity of sounds that can be produced by biological systems with coupled oscillators in the animal kingdom, allowing species to convey different types of information through their vocalizations.

## 5. Predictions from oscillator theory

Normal sustained phonation in the context of vocal production can be understood through limit cycles. Here, vocal folds oscillate in a stable, self-repeating pattern owing to a balance between energy input and dissipation. Energy, derived from respiration and muscle activity, compensates for the inherent dissipation caused by tissue resistance and airflow dynamics. This energy is needed to stabilize the amplitudes and frequencies of vocal fold vibrations, which otherwise would dampen to silence. Two other ingredients are needed in a dynamical system to generate self-sustained limit cycle oscillations [19]: nonlinearities and negative feedback loops. In animal vocalization, examples of nonlinearities are vocal fold collisions and aerodynamic forces. Collisions disturb the harmonic vibrations of vocal folds and interrupt airflow. The Bernoulli equations connect airflow and pressure and are intrinsically nonlinear [16].

Negative feedback mechanisms are equally important because such network schemes are needed to carry the system back to where the oscillation started. Negative feedback loops are circular systems where a variable is negatively affected by its interaction with other variables. They can result in a delayed response that helps stabilize the oscillating system by opposing rapid changes. According to Newton’s laws of motion, mechanical systems are accelerated or decelerated in response to forces. Because acceleration is the second derivative of position (with respect to time), and the second derivative of an oscillation like sin⁡(t) is −sin⁡(t), it becomes evident that second-order time derivatives can act as negative feedback loops. In the context of vocalization, restoring forces such as tissue elasticity play a crucial role, providing feedback that returns the system to its original state after each oscillatory cycle. Together, nonlinearities and negative feedback schemes ensure that vocal fold oscillations remain stable, contributing to the clarity and stability of phonation.

The myoelastic–aerodynamic theory [16] provides a framework to explain and understand how most of the animal vocalizations are generated. According to this theory, the interaction between muscle tension, tissue elasticity and airflow dynamics governs vocal fold vibration. Airflow through the vocal tract provides the necessary energy to sustain and regulate these vibrations, ensuring their stability and facilitating the production of sound. However, additional factors can contribute to the complexity of phonation. Asymmetries in vocal fold structure or tension [27,43,60] can introduce variations in sound production and generate NLP, including subharmonics, biphonation or chaos. Moreover, anatomical structures such as vocal membranes [10,82] can also influence how vibrations propagate through the vocal system and can result in the emergence of NLP. Furthermore, different vibration modes along the anterior–posterior axis of the vocal folds [62,64], or interactions of the vocal fold vibrations with oscillating air columns, such as whistles or air sacs [12,61] also contribute to the richness and diversity of vocal sounds. Additionally, neuronal feedback mechanisms can lead to phenomena like voice tremor and vibrato [83,84], adding further complexity to the mechanisms of vocalization. Together, these factors illustrate the intricate interplay of physiological and biomechanical elements involved in vocal production and generation of NLP.

Despite the anatomical and physiological complexities underlying sound production, the repertoire of NLP remains relatively small, restricted to limited cycles, frequency jumps, subharmonics, chaos, biphonation and modulations. As we discussed in §3, limit cycles can be destabilized via period-doubling or secondary Hopf bifurcations, producing subharmonics and tori, respectively. Subcritical bifurcations with hysteresis can lead to frequency jumps, with a parameter range in which two limit cycles coexist (as has been shown, for example, in songbirds like the common nightingale, which can switch between loud and soft calls [85], similar to human transitions between chest voice and falsetto [44,45]). Humpback whales [86] or killer whales [66] are known for their complex songs, which involve varying pitch and tonal qualities, akin to human vocal modulation. Finally, a cascade of period-doubling bifurcations can also generate deterministic chaos [46] (the most spectrally complex of all NLP) for very high coupling. Oscillator theory therefore predicts that the chaotic dynamics characteristic of tiger roars [87], crocodiles [32] or newborn cries [33] require highly coupled vocal oscillations.

The interaction between vocal fold oscillations (the source) and vocal tract resonances (the filter) is another potential source of NLP. Source–filter interaction describes how the sound produced by vocal folds (the source) is shaped by the vocal tract (the filter) [88]. In a linear interaction, the source and filter are decoupled, with the vocal folds producing a sound that is filtered passively by the vocal tract. The source and filter act independently, producing a harmonic spectrum with no influence of the filter on the source’s behaviour [88]. However, for narrow epilaryngeal tubes and similar frequencies of the source and filter, a reasonable nonlinear coupling can occur [50]. This nonlinear source–filter interaction involves significant feedback between the two, where acoustic pressures in the vocal tract can actively affect the vocal fold oscillations. This two-way interaction leads to complex phenomena where subglottal pressure or vocal fold tension can induce bifurcations, resulting in frequency jumps, subharmonics, biphonation or chaotic regimes [50,89]. These complex source–filter interactions further illustrate the inherently nonlinear nature of voice production in both human and animal vocalizations.

As described in §4, Arnold tongue diagrams organize the NLP and offer insights into how parameter changes control the dynamical behaviour. Weak coupling often results in toroidal oscillations, observed in vocalizations from dogs and other canids [35,90,91] or in horses [12] and other ungulates including deer and elks [6,31]. According to the bifurcation theory of Arnold tongues, increasing coupling in such toroidal oscillations will predict systematic transitions to frequency locking and finally to chaos through a period-doubling cascade (figure 6, movement along vertical lines). Similarly, if for medium coupling strength K frequencies are detuned progressively, oscillator theory predicts transitions from 1 : 2 rhythms to tori, to 2 : 3 frequency locking and finally, to a 1 : 1 synchronization (movement along horizontal lines in figure 6). Overlapping tongues of frequency-locked regions induce frequency jumps with hysteresis, akin to register transitions between chest voice and falsetto [42,44,45,74,92]. Taken together, bifurcation diagrams and Arnold tongues of coupled nonlinear oscillators help in understanding and explaining the generation of NLP in animal and human bioacoustics. Studying the underlying oscillators and the different ways in which these vibrating structures can interact is critical to understand the physiology of vocalization as well as the evolutionary pressures that have shaped communication strategies in different species.

## 6. Possible functions of nonlinear vocal phenomena

Coupled oscillators in the context of vocalization naturally exhibit a variety of NLP. These NLP can manifest as complex acoustic patterns, such as biphonation, subharmonics, chaos and other intricate spectral and temporal features that deviate from simple periodic oscillations. The emergence of NLP in vocalizations arises as by-products of high intensities or asymmetries in the vocal systems of various organisms. For example, high-amplitude vocalizations, like loud roars or screams, can be owing to high mechanical or aerodynamic coupling between the vocal folds or other sound-producing structures. Similarly, asymmetries in the vocal tract or the coupling between different oscillating components can lead to the generation of other NLP. Despite initially arising as by-products of intense or asymmetric vocal systems, natural selection has exploited the inherent complexity of these vocal mechanisms over evolutionary timescales. The presence of NLP in animal vocalizations can confer various adaptive advantages, such as encoding individual identity [4,91,93], mating availability [94], signal arousal or fear to receivers [95], conveying information about body size [76] or dominance status.

Animal vocalizations often encode individuality through intricate dependencies among various parameters. Minor anatomical and physiological variations can lead to distinct vocal signatures. For instance, it has been proposed that the variability in bank swallow calls allows for individual identification among large populations of birds [96]. Similarly, the prevalence of biphonic calls in penguins also allows for individual recognizability between mothers and their chicks [4]. Evidence also supports individual distinctiveness in vocalizations among dholes [91].

Female koalas produce vocal signals when they reject male copulation attempts that contain high levels of NLP, being particularly rich in subharmonics [94]. These rejection calls, in turn, incite male-to-male competition during the breeding season and are thought to function to ultimately grab male attention [94]. In other species, including marmots, animal vocalizations can abruptly become ‘noisy’ and rich in NLP when individuals are physiologically aroused. Sudden onset of NLP has been demonstrated to signal arousal or fear to receiver marmots through playback experiments, where alarm calls rich in NLP elicited a greater response than control calls [95].

Broadband signals can also convey information about body size through formant spacing [97]. In speech science and phonetics, formants are prominent bands of frequency that determine the phonetic quality of a vowel, resulting from acoustic resonance of the human vocal tract. Consequently, loud roars and screams containing many NLP may be used to signal dominance. Subharmonics and modulations have been suggested to be used by animals to suggest a larger body size [76].

Arnold tongue diagrams visually represent the complex behaviour of coupled oscillators (figure 6), such as those found in vocalization. These diagrams illustrate the relationships between different parameters and the resulting NLP, bifurcations (sudden changes) and attractors. The complexity depicted in these diagrams suggests that precisely controlling and manipulating parameters to regulate the generation of specific NLP might be challenging. However, despite this difficulty, the mere presence of NLP in vocalizations may already serve an important function by enhancing the attention of listeners [90,94,98]. These inherent attention-grabbing qualities might have evolved as an adaptive advantage, regardless of the ability to control them. In other words, the presence of NLP in vocalizations might be an evolutionary trait that enhances the impact of the vocalization of listeners, even if the vocalizer cannot consciously manipulate or control the specific NLP involved. For instance, newborn cries particularly attract attention from their mothers [34]. Recent findings also indicate that the frequency of NLP in dog puppy whines increases with separation from the mother [93], and that begging calls from hatching penguin chicks also contain NLP—mainly sidebands and chaos [99], which might serve to capture the attention of the adults’ attention.

Interestingly, also the absence of NLP might be of adaptive value and might convey information about how stable the vocal source is. In the animal kingdom, there are situations where the vocalization is normally harmonious and consequently with low content of NLP. For instance, loud vocalizations such as panhoots in chimpanzees or mating calls in the North American elk have been suggested to be designed to produce a very high fundamental frequency with maximized intensity in what has been termed ‘vocalizing at the edge’ in the literature [100]. It has been suggested that animals that call a lot and very loudly (with high intensity of the fundamental frequency), for example during the mating season, are less rich in NLP. Conversely, less-dominant male primates that have been subjected to a chase event and show signs of exhaustion, typically show more frequent occurrence of NLP [101,102].

While it is difficult to prove the exact functions of NLP and why they have evolved in the way they have, an interdisciplinary approach using both experimental and computational methods might provide some answers and allow for speculation on the possible adaptive functions of nonlinear vocal phenomena.

## 7. Concluding remarks

Nonlinear dynamics offers a robust theoretical framework for understanding the complex behaviour of dynamical systems, providing valuable insights into the underlying mechanisms that govern the occurrence of attractors and bifurcations. Given the inherently nonlinear nature of voice production in humans and animals, which can be modelled as a system of coupled oscillators, the tools and theory of nonlinear dynamics are highly relevant and applicable in the field of bioacoustics.

In the study of vocalizations (and any oscillating system), it is essential to distinguish between transients and attractors. Attractors, such as limit cycles, tori and deterministic chaos (figure 2), are relevant for relatively stationary signals like newborn cries, sustained vowels and steady animal vocalizations. Transients become equally important in the analysis of non-stationary signals, including muscle activity that results in speech in humans or birdsong. For example, ultrafast muscle activations within milliseconds can resemble subharmonic bifurcations in birdsong [103]. Computer simulations have been shown to be a valuable tool and can be exploited to study transients in birdsong [60] and patients [43].

This article focused on bifurcations, which are qualitative changes in system behaviour owing to parameter variations, and the attractor that emerges as a result of it. Relevant bifurcations in bioacoustics include Hopf bifurcations (figure 3), period-doubling bifurcations (figure 4) and frequency jumps between limit cycles (figure 2E). These bifurcations can occur smoothly (e.g. supercritical Hopf bifurcation) or abruptly (e.g. subcritical Hopf bifurcation, as in hard phonation onset). Sudden transitions between limit cycles have been extensively studied in the context of voice registers [44,92,104,105] and observed in excised larynx experiments [26,42,74].

We also discussed Arnold tongue diagrams, which provide a comprehensive visualization of attractors and the different dynamical states that are possible among coupled oscillators [46]. These two-dimensional bifurcation diagrams, with frequency ratio and coupling strength as governing parameters, help in structuring the complex organization of frequency-locking regions, tori and deterministic chaos (figure 6). Studying Arnold tongue diagrams is of relevance because it can hint at features of the underlying vocalizations. For example, the frequent observations of biphonations in horse vocalizations [12] suggest weakly coupled voice-generating sources. Frequency jumps with hysteresis (a phenomenon where the system’s behaviour depends on its previous state, figure 3B) can be expected when frequency-locking regions start to overlap. Series of period-doubling bifurcations associated with subharmonics often precede deterministic chaos.

In summary, by combining the theoretical frameworks of nonlinear dynamics, computer simulations and careful observations of animal vocalizations, researchers can potentially gain a deeper understanding of the adaptive functions of NLP. This synergistic approach, which integrates theoretical models, computational tools and empirical data, can shed light on the evolutionary pressures and adaptive advantages that have shaped the richness and complexity of vocalization mechanisms and communication strategies across different species.

## Data Availability

This article has no additional data.
